# An interdisciplinary statement of scientific societies for the advancement of delirium care across Europe (EDA, EANS, EUGMS, COTEC, IPTOP/WCPT)

**DOI:** 10.1186/s12877-019-1264-2

**Published:** 2019-09-11

**Authors:** Alessandro Morandi, Christian Pozzi, Koen Milisen, Hans Hobbelen, Jennifer M. Bottomley, Alessandro Lanzoni, Verena C. Tatzer, Maria Gracia Carpena, Antonio Cherubini, Anette Ranhoff, Alasdair M. J. MacLullich, Andrew Teodorczuk, Giuseppe Bellelli

**Affiliations:** 1 Department of Rehabilitation and Aged Care “Fondazione Camplani” Hospital, Cremona, Italy; 2 Rehabilitation Hospital Ancelle di Cremona, Via Aselli 14, 26100 Cremona, CR Italy; 3 University of Applied Sciences and Arts of Southern Switzerland (SUPSI), Manno, Switzerland; 4 Department of Public Health and Primary Care, Academic Centre for Nursing and Midwifery, KU Leuven, Leuven, Belgium; 5 Division of Geriatric Medicine, University Hospitals Leuven, Leuven, Belgium; 6 Healthy Lifestyle, Ageing and Healthcare Research group Healthy Ageing, Allied Health Care and Nursing Centre of Expertise Healthy Ageing Hanze University of Applied Sciences Groningen, Groningen, Netherlands; 7 International Physical Therapist Working with Older People, Subgroup of World Confederation for Physical Therapy, WCPT, London, UK; 8 Simmons College, Boston, MA USA; 9 NODAIA Unit, Villa Igea, Modena, Italy; 10 University of Modena and Reggio Emilia, Modena, Italy; 11 University of applied Sciences Wiener Neustadt, Neustadt, Austria; 12 Center Superior de Estudios Universitarios La Salle (UAM), Madrid, Spain; 13 Geriatria, Accettazione geriatrica e Centro di ricerca per l’invecchiamento, IRCCS INRCA, Ancona, Italy; 14 Department of Clinical Science, University of Bergen, Bergen, Norway; 15 Edinburgh Delirium Research Group, Geriatric Medicine, Division of Health Sciences, School of Clinical Sciences, University of Edinburgh, Edinburgh, UK; 16 Centre for Cognitive Ageing and Cognitive Epidemiology, University of Edinburgh, Edinburgh, UK; 17 School of Medicine, Gold Coast Campus, Griffith University, Mount Gravatt, QLD Australia; 18 School of Medicine and Surgery, University of Milano-Bicocca, Milan, Italy; 19 Geriatric Unit, S. Gerardo Hospital, Monza, Italy

**Keywords:** Delirium, Interdisciplinary collaboration, Physical therapy, Occupational therapy

## Abstract

**Background:**

Delirium is a geriatric syndrome that presents in 1 out of 5 hospitalized older patients. It is also common in the community, in hospices, and in nursing homes. Delirium prevalence varies according to clinical setting, with rates of under 5% in minor elective surgery but up to 80% in intensive care unit patients. Delirium has severe adverse consequences, but despite this and its high prevalence, it remains undetected in the majority of cases. Optimal delirium care requires an interdisciplinary, multi-dimensional diagnostic and therapeutic approach involving doctors, nurses, physiotherapists, and occupational therapists. However, there are still important gaps in the knowledge and management of this syndrome.

**Main body:**

The objective of this paper is to promote the interdisciplinary approach in the prevention and management of delirium as endorsed by a delirium society (European Delirium Association, EDA), a geriatrics society (European Geriatric Medicine Society, EuGMS), a nursing society (European Academy of Nursing Science, EANS), an occupational therapy society (Council of Occupational Therapists for European Countries, COTEC), and a physiotherapy society (International Association of Physical Therapists working with Older People of the World Confederation for Physical Therapy, IPTOP/WCPT).

**Short conclusion:**

In this paper we have strongly promoted and supported interdisciplinary collaboration underlying the necessity of increasing communication among scientific societies. We have also provided suggestions on how to fill the current gaps via improvements in undergraduate and postgraduate delirium education among European Countries.

## Background

Delirium is a geriatric syndrome characterized by an acute change and fluctuation of cognitive function, inattention and impaired awareness [1]. Delirium is a multifactorial condition that does not fit the traditional disease model. There are several predisposing factors, such as dementia, malnutrition, and sensory impairment, and it is generally triggered by medical causes, pain and/or drugs [1, 2]. It occurs on average in one out of 5 hospitalized older patients [3]. Although the majority of studies have been performed in the hospital, delirium is not limited to geriatric wards or a geriatric inpatient population. Delirium prevalence in hospitals varies according to the clinical settings. It ranges from less than 5% in with some elective surgery, 18 to 35% in medical/geriatric wards and up to 80% in Intensive Care Units (ICUs) [4]. The highest incidence is observed in ICUs and in palliative care settings [4]. Delirium is also common in nursing home residents and in postacute settings, with a prevalence ranging from 1.4 to 70.3% [5], and between 13 and 26%, respectively [6, 7]. The presence of delirium at home is relatively low with a prevalence ranging from 1 to 2% [4, 8]; though a recent study has reported a prevalence as high as 13% in an outpatients memory clinic [9]. However, few studies have addressed delirium epidemiology outside the hospital thus limiting our ability to provide detailed information of delirium prevalence in other settings.

Delirium has several prognostic implications including worsening of cognitive and functional status [10, 11], increasing patients’ and caregivers’ burden [12, 13] and elevated mortality in the middle-long term [4, 14]. A robust association has been demonstrated between delirium and in-hospital mortality in the ICU [4, 15, 16]; few studies have assessed the association of delirium with in-hospital death in medical and surgical wards [17, 18]. Delirium is also associated with a significant increase in health care costs [19]. Additionally, it is now well recognized that when delirium persists the outcomes are even worse, underlying the importance of an early recognition and a daily assessment [20]. Indeed, each additional day of delirium has been described to be associated with a 10% increased in mortality at 6 months in different clinical settings [15, 21].

Despite its high prevalence, delirium is unrecognized in the majority of cases, especially when a specific tool is not used for screening [22–24]. Underdetection has been linked associated with increased mortality [25]. The increased mortality related to underdetection might be due to delirium itself or to the lack of recognition and treatment of the underlying causes or both. Delirium has been, therefore, recognized as a medical emergency given its outcomes [4]. It is imperative to screen for delirium using validated tools and when delirium is recognized a thorough assessment should be carried out to determine the precipitating factors.

The most recent evidence-based guidelines, published in March 2019, and recent consensus statements recommend the prioritization of multicomponent nonpharmacological approach for the prevention and treatment of delirium [26, 27]. The guidelines suggested that there initially should be a non-pharmacological approach, identifying and managing underlying causes, providing reorientation and involving family and carers. Primary prevention of delirium with non-pharmacological multicomponent interventions is effective [28–30]. Specifically there is strong evidence supporting a reduction of the incidence of delirium compared to usual care (Relative Risk 0.69, 95% CI 0.59 to 0.81) [28, 29]. The findings are similar in medical and surgical settings, while in the subgroup of patients with pre-existing dementia, the effect of multi-component interventions remains uncertain [28, 29]. In contrast, another meta-analysis showed that multicomponent interventions significantly reduced incident delirium (relative risk, RR 0.73, 95% confidence interval CI 0.63–0.85, *P* < 0.001), without evidence of differential effectiveness according to ward type or dementia rates [31]. The multicomponent intervention adopted includes reorientation, drugs reconciliation and reduction of psychoactive drugs, promotion of sleep, early mobilization, adequate hydration and nutrition, use of vision and hearing devices. It requires an interdisciplinary approach including physicians, nurses, physical and occupational therapists along with trained family member and/or trained volunteers. Although it is now recognized that the multiprofessional approach is key in the management of delirium there is still a wide gap on the knowledge and education concerning this geriatric syndrome. Previous surveys have mainly addressed the knowledge of delirium among geriatricians and nurses and only one survey has included a limited group of physical therapists [32–34]. Only, recently the application of occupational therapy has been specifically studied for delirium care [35–37].

This paper will focus on 1) promoting, via the support of scientific societies, the interdisciplinary collaboration of physicians, nurses, physical and occupational therapists, although it is acknowledged that other members of staff could and should be involved in delirium care, i.e. clinical pharmacists, nutritionists, nursing assistants and, when available, family members and caregivers; 2) underlying the current obstacles of delirium care; and 3) approaching ways to improve delirium care across Europe.

### The interdisciplinary approach (geriatrics, nursing, occupational therapy, physical therapy) in advancing delirium care, awareness and knowledge

Different geriatric care models based on a comprehensive geriatric assessment (CGA) have been developed in the last thirty-five years to improve geriatric patients’ outcomes [38]. Specifically, older adults are more likely to be alive and in their own homes at follow-up if they received CGA on admission to hospital [38].

These models include an interdisciplinary, multi-dimensional diagnostic and therapeutic process involving geriatricians or other medical clinicians, nurses, physiotherapists, occupational therapists, speech therapists, nutritionists, clinical pharmacists and social workers. This comprehensive assessment leads to a coordinated and integrated plan for treatment and follow-up [39]. However, it has been recently reported that the implementation of multifaceted and interdisciplinary interventions might indeed be challenging and it is necessary to better describe, disseminate and expand evidence-based geriatric models [40].

The occurrence of delirium can be seen as a proxy measure of quality of inpatient care in older patients and effective interventions for delirium prevention have to be considered integral to quality improvement [4]. Focusing on delirium prevention may help developing the necessary multiprofessional skills, cultural aspects, and service design to improve the quality of care. The Assessing Care of Vulnerable Elders Project has ranked delirium among the top three conditions for which the quality of care needs to be improved [41].

Effective interventions to enhance delirium management should go beyond the education of a single profession and instead adopt an interdisciplinary approach, including multiple health professions that learn with and from and about one another [42]. It has been shown how interprofessional practice changes combined with interprofessional education may influence synergistically team and patients outcomes [42, 43]. Moreover, new educational methods (e.g. simulation of delirium performed by actors) might be valuable in improving knowledge and understanding [44].

It is well known that delirium is a complex geriatric syndrome often caused by multiple coexisting etiological factors including for instance malnutrition, dehydration, use of physical restraints, iatrogenic events and polypharmacy. The interdisciplinary team will be involved in a collaborative manner to manage the complexity addressing immobility, hypoxia, poor nutrition and dehydration, constipation, cognitive impairment and sensory impairment. Specific tasks might be limited to the geriatrician and the nurse such as medication review to optimize polypharmacy, the treatment of infections and sleep disturbances. Hence interprofessional education is key to developing effective collaborative practice approaches.

Though nursing involvement in the management in delirium care has been widely described in different interventions [29], only recently more attention has been given to the contribution of physiotherapists and occupational therapists. An important study has underlined how an early physical and occupational therapy intervention might indeed reduce the sequelae related to delirium [37]. Among the most powerful interventions to reduce the risk for delirium are exercise and early mobilization guided by a physiotherapist. A recent systematic review of randomized clinical trials showed that it is feasible to provide exercise and early rehabilitation in hospitalized older adults [45]. Moreover a recent study in cardiac surgery patients suggests that preoperative exercise to enhance exercise capacity can prevent delirium after surgery [46]. However, it is challenging for health care providers and policy makers to change an approach based on the care of the disease to one focused on the person, with the main goals to optimize functional and cognitive recovery after hospitalization. The American Geriatric Society has provided the base to support a definition of person-centered care among with the essential element to realizing this approach including the following items “1) an individualized, goal-oriented care pla based on the person’s preferences; 2) ongoing review of the person’s goals and care plan; 3) care supported by interprofessional team; 4) a lead point to contact on the healthcare team; 5) active coordination among all healthcare providers; 6) continual information sharing and communication; 7) education and training for providers, the person and those important for the person; 8) performance measurement and quality improvement using feedback from the person and the caregivers” [47].

Other studies have shown how occupational therapy treatment combined with a standard non-pharmacological protocol is effective in reducing the duration and incidence of delirium [35, 36]. The OT treatment is focused on improving autonomy and on increasing the sense of efficacy, satisfaction and well-being of patients using everyday activities as means and goals of the treatment. It is important to underline that the main outcome of the OT is the occupational performance: it means that the activity subjected to the treatment has to be performed as best as possible, regardless of its subsystems [48]. This premise suggests that occupational therapy could be useful in the treatment of people with delirium because it aims to improve ADL by offering suggestions for medical devices, problem solving strategies and support and advice to caregivers [49]. The OT must adjust and modify activities depending on the functional and cognitive skills of the patient in collaboration with the nurses and the physiotherapists. The treatment requires a collaborative collection of an adequate background information about the history of the patient because the rehabilitative process has to be designed based on the patients’ familiar and professional roles, of essential events in their life, and their tastes, inclinations, values and beliefs. The nurse, the PT and the OT must know the most important delirium evaluation scales (e.g. 4AT, DOS, m-RASS etc.) [50–52] in order to be flexible during the treatment and understand the persistence or the resolution of delirium. Then each individual in the interdisciplinary team will use a specific scale to monitor their intervention. For instance the OT has specific scales to identify the goals (e.g. COPM in case of temporary or moderate degenerative deficit [53]; Model of Human Occupation, occupational engagement [54]; model of take in charge as Allen Cognitive Disabilities Model), to measure the occupational performance (Assessment Motor and Process Skills [55]; to observe in detail occupational skills, personal motivation and relationship with the environment (Volitional Questionnaire) [56]. The PT would choose specific scales to monitor motor functions and basic mobility such as the Performance-oriented mobility assessment (i.e. Tinetti Scale), the de Morton Mobility Index (DEMMI), the Trunk Control Test (TCT), the Shorth Physical Performance Battery (SPPB), and the HABAM scale [57–61]. The Barthel Index would be frequently used as a common scale to monitor the overall functional status of the person with delirium [62]. In particular, specific Barthel Index sub-items (i.e., walking, transfer, dressing) can be shared by all the figures and monitored throughout the treatment.

According to the current evidence we summarize in Table 1 the main goals of the interdisciplinary intervention of nurses, physiotherapists and occupational therapists to maximize collaboration.

**Table 1:** Goals and interventions of the interdisciplinary collaboration between nurses, occupational therapists, and physiotherapists

Goals	Interventions
Improvement of the autonomy and involvement in everyday activities	1) Creation of a meaningful routine that alternate activities and rest periods, promoting a 24 h rehab vision and fighting occupational deprivation; 2) Promptly set up mobility as changing of posture (supine/seated), changing of sleeping posture and suspend bed-blocking as soon as possible; 3) Promotion of mobility allowing the patient to interact functionally with the environment: B/ADL activities in bathroom, meals seated at the table, play games (e.g., Sudoku or cards).
Environment adaptation	Conform the environment to the need of the person suffering of delirium: reduction of disperceptive sensory stimuli, softening of the noises, appropriate lighting, reduction of sensory deprivation.
Evaluation of assistive devices	Selection of the best devices in order to safeguard an appropriate posture in bed, on the chair and/or in wheelchair.
Family education	1) Preparing family caregivers to recognize delirium symptoms 2) Favor a proactive presence of the family (human environment) teaching them how to approach and how to communicate with the patient in order to decrease agitation in older hospitalized delirious patients

### Current obstacles in advancing delirium care across Europe

**Fig. 1:**
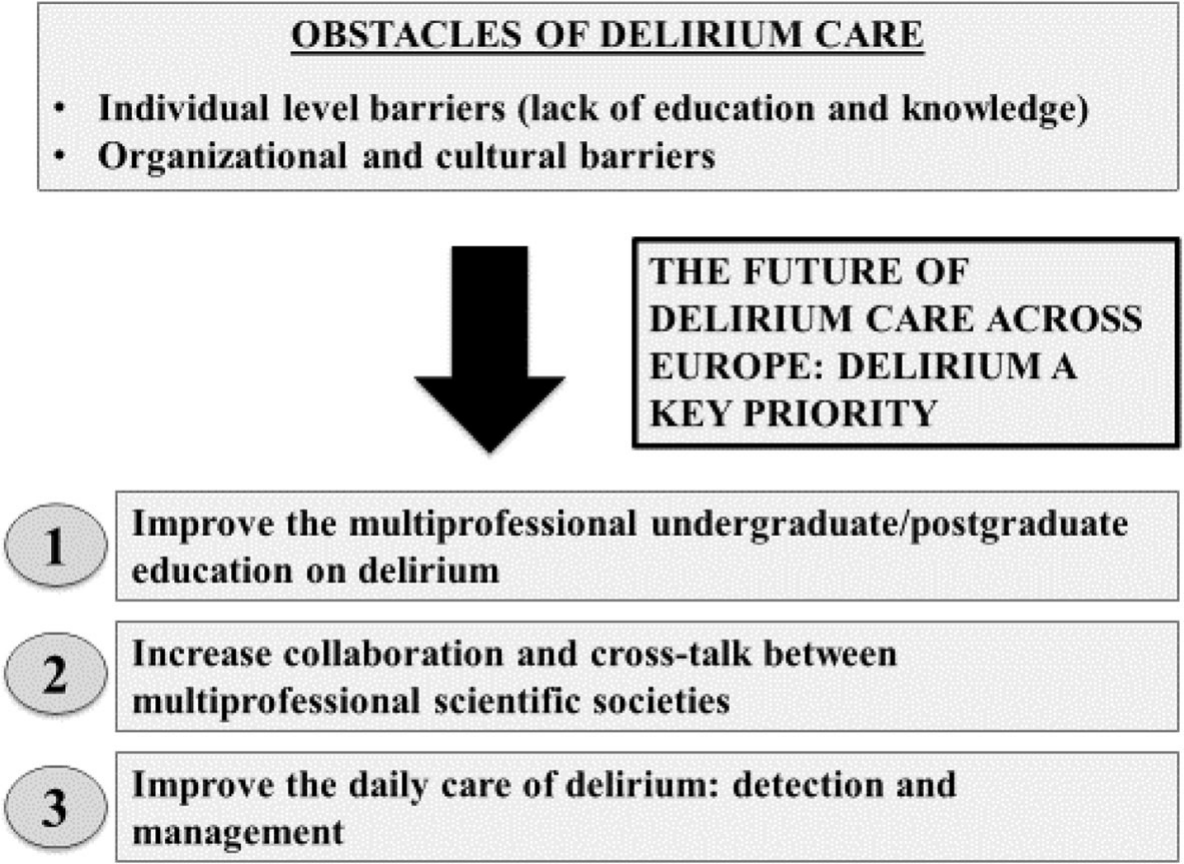
The future of interdisciplinary delirium care across Europe

Unfortunately, there is growing evidence of persistent obstacles in delirium care across Europe including a) attitude, culture, and language; b) knowledge, skills and education; c) organization (Fig. 1). A recent international survey among delirium experts within the European Countries reported that low delirium awareness, inadequate knowledge/incompetence, lack of education, and lack of time for assessment were identified as the four main barriers to improving delirium detection [33]. Similarly, poor knowledge, staffing issues, poor education, and poor attitudes were the main barriers to improving delirium management [33].

#### Individual level barriers

After conducting workshops within two EDA meetings in 2010 and 2011, Teodorczuk and colleagues [63] specifically focused on exploring the main barriers to improve the detection of delirium and proposed a “call for action” plan involving individual, organizational, and societal level changes to improve recognition. They identified *“individual level barriers”* as an overall lack of education and awareness and further identified a high degree of uncertainties on the benefits of delirium recognition and treatment [63]. It was further reported how there is often poor knowledge of delirium among trainee doctors [34], despite the fact that a recent survey exploring delirium teaching among UK medical schools has reported that delirium is widely taught and assessed [64]. This might partly be explained by the fact that the schools tend to focus on knowledge and skills, with limited attention on attitudes.

This reported knowledge gap has implications not only for doctors but for all professionals involved in undergraduate and postgraduate educations of doctor, nurse, physiotherapists and occupational therapists. Indeed a recent Italian survey involving physiotherapists and nurses along with doctors found there was a small proportion of physiotherapists and nurses who actually used a specific tool to diagnose delirium and only half of them correctly defined it [32]. Given the importance of a collaborative multiprofessional approach, competence in diagnosing and managing delirium should not be limited to doctors, but we should strive to expand delirium recognition in nurses, physiotherapists and occupational therapists. Indeed, it has now been shown in different publications that there is an association between delirium and functional status [65, 66]. Specifically, a rapid change in functional status has been reported to be a prodromal sign of delirium in frail patients. Therefore, physiotherapists and occupational therapists would have a central role in delirium recognition and treatment. Crucially, nursing staff and health-care assistants spend greater periods of time with hospitalized in-patients than medical staff and their insight and knowledge of the patient and carer is frequently critical to delirium recognition [67].

#### Organizational and cultural barriers

It has been also reported how there are key *organizational and cultural barriers* to good delirium practice [63]. Delirium is often not perceived as a priority and people believe that delirium does not belong to a specific specialty, making it an orphan syndrome. For instance, the World Health Organization (WHO) report on Ageing and Health only briefly mentions delirium as a geriatric syndrome while other complex health conditions such as frailty, urinary incontinence, and falls are discussed more in detail [68]. However, it underlines how older age is also characterized by the emergence of several complex health states that tend to occur only later in life and that do not fall into discrete disease categories. Delirium, indeed, as a geriatric syndrome, should be a priority for geriatric scientific societies and related societies of other professional parties as nurses, physiotherapists, and occupational therapists. There is also a lack of public awareness for delirium and the scientific societies should further work on initiatives such as a National Delirium Day [3] and World Delirium Awareness Day (http://www.idelirium.org/) to fill this gap.

Another important point, which should be considered, is the cost-effectiveness of a multiprofessional approach. As previously highlighted, studies have shown how a multiprofessional intervention is effective in preventing delirium [69]. Rubin and colleagues have reported the sustainability and scalability of the Hospital Elder Life Program (HELP) with a significant yearly cost saving in the management of older patients with delirium [70]. A subsequent study confirmed the possibility to translate this program into daily clinical practice [71]. However, the HELP program includes the intervention of volunteers, who are not always available in the clinical settings. More studies are warranted to further investigate the sustainability and the cost-effectiveness of a multiprofessional team in the daily clinical practice, in settings/countries where the healthcare staff is in charge of implementing the intervention.

### The future of delirium care in Europe

#### Multiprofessional under- and postgraduate education process

In the last years supporting (e.g, use of reminders) and empowering (e.g., use of guidelines, or protocols) strategies have been shown to be efficient in improving the care of delirium, with direct effect on delirium outcomes [72–74]. However, the implementation and the ability to maintain the adherence to these educational initiatives is time consuming and labour intensive [75]. Educational activities should be repeated at regular intervals in healthcare settings where delirium prevention and management are implemented, due to the high turnover of staff, in particular but not exclusively nurses.

E-learning has been described as a novel approach to facilitate the possibility to provide education for large groups of people. Specifically, an e-learning program on delirium of 11 modules has been proven to be effective in improving the knowledge and recognition of delirium [76]. However, to improve uptake and effect on patient outcomes, further research is warranted to explore the efficacy of delirium e-learning programs along with educational initiatives including supporting and empowering strategies [77].

Attitudes to delirium practice have also been described as a central barrier to good practice [78]. Coupled with a lack of ownership of the patient, arguably because of the greater complexity posed, the narrative on the ward often may shift from a therapeutic clinical relationship to one where the team will seek to remove the patient from the ward. One approach to tackle this would be to address and shape attitudes early by teaching delirium effectively at a prequalification stage. A recent publication on effective curriculum development clearly defined through a Delphi approach how, by whom, where and when should undergraduate medical delirium teaching be delivered [79]. Geriatricians, old age psychiatrists, and nurses have to be involved in delirium teaching among with family members and patients as well. Moreover, teaching should be delivered by an interdisciplinary team in the context of acute setting and long-term care and in the early years of an undergraduate curriculum. The curriculum should focus on three main areas of delirium: 1) etiology, epidemiology, and pathophysiology; 2) diagnostics; 3) management. For each of these points the authors describe in detail upon what they agree should be thought and what the students are expected to learn during the training. It should be noticed that during the Delphi approach a number of specialties (e.g. palliative care, pediatrics) were removed from the figures involved in delirium education due to a failure to achieve consensus in this area. Additionally, the authors underline the importance of a multiprofessional approach in delirium teaching, since interprofessional education may positively influence team and patient outcomes in delirium care [42].

#### Collaboration among health care professionals

As it has been highlighted, delirium is not only limited to a geriatric population or geriatric wards but it is present in different clinical settings. Given the outcomes related to delirium and especially its underrecognition, it is essential that all health care professionals have adequate knowledge of this syndrome. In this paper we strongly support the formation of an interdisciplinary approach in the management of delirium involving a specific delirium society (European Delirium Association, EDA; http://www.europeandeliriumassociation.com), a geriatric society (European Geriatric Medicine Society, EuGMS; http://www.eugms.org/home.html), a nursing society (European Academy of Nursing Science, EANS; https://european-academy-of-nursing-science.com); an occupational therapy society (Council of Occupational Therapists for European Countries, COTEC; http://www.coteceurope.eu); and a physiotherapy society (World Confederation for Physical Therapy, WCPT; http://www.wcpt.org and its official subgroup The International association of Physical Therapists working with Older People IPTOP; https://www.wcpt.org/iptop/about). Similar to pushing for greater collaboration amongst disciplines on the ward by proposing a unified approach organizational level it is likely that system barriers may be more likely to be overcome.

#### Improve the daily clinical practice: screening, diagnosis, surveillance, monitoring for recovery

**Fig. 2:**
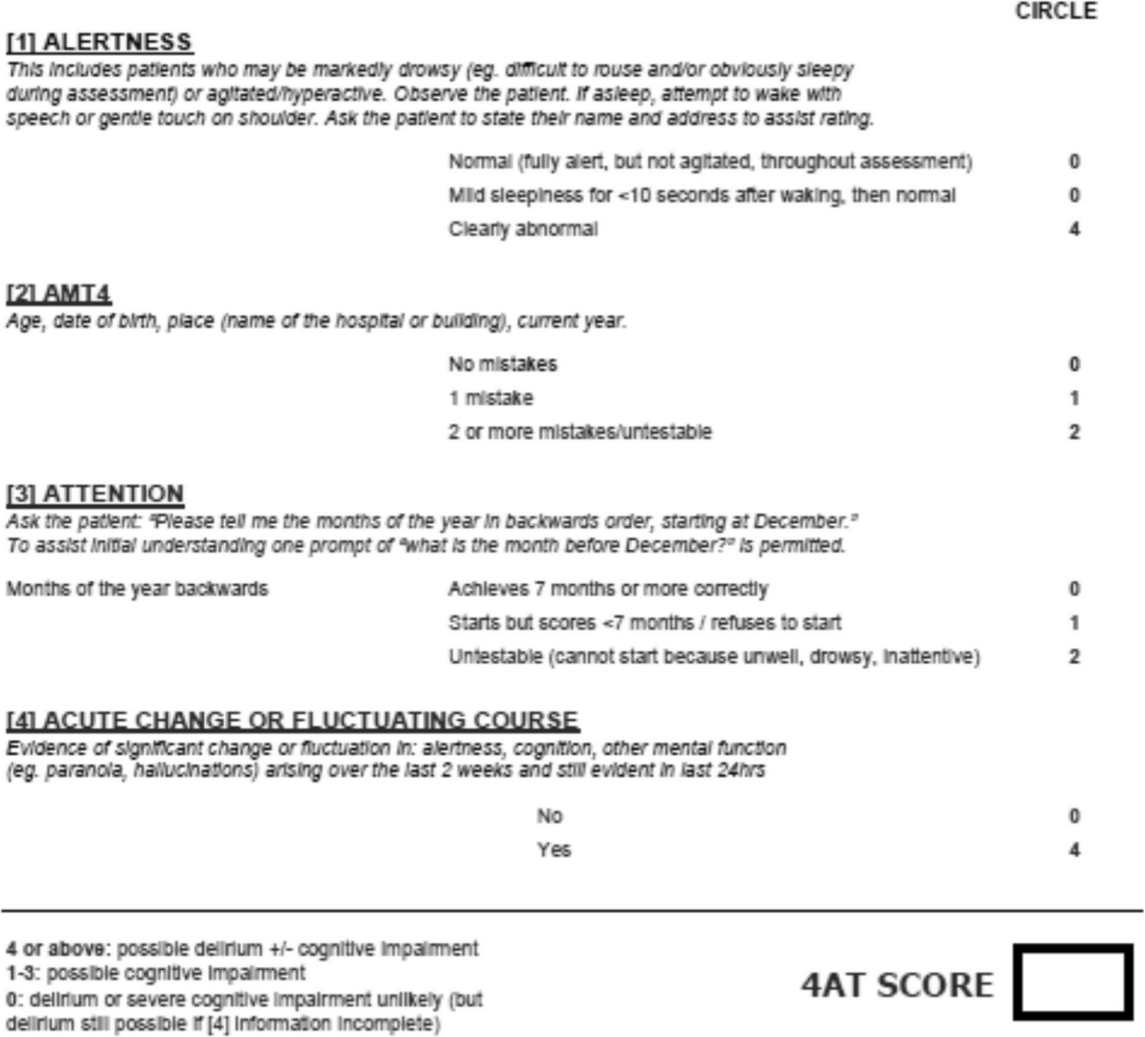
4AT assessment test for delirium. The 4AT is a screening instrument designed for rapid initial assessment of delirium and cognitive impairment. A score of 4 or more suggests delirium but is not diagnostic: more detailed assessment of mental status may be required to reach a diagnosis. A score of 1–3 suggests cognitive impairment and more detailed cognitive testing and informant history-taking are required. A score of 0 does not definitively exclude delirium or cognitive impairment: more detailed testing may be required depending on the clinical context. Items 1–3 are rated solely on observation of the patient at the time of assessment. Item 4 requires information from one or more source(s), e.g. your own knowledge of the patient, other staff who know the patient (e.g. ward nurses), GP letter, case notes, carers. The tester should take account of communication difficulties (hearing impairment, dysphasia, lack of common language) when carrying out the test and interpreting the score

The diagnosis of delirium currently relies on the diagnostic criteria of the Diagnostic and Statistical Manual of Mental Disorders-5 (DSM-5) and the International Classification of Disease-10 (Table 2) [80, 81]. However, it is well recognized how these criteria could limit delirium screening and diagnosis since they require extensive training to be applied. In recent years, several tools have been developed for delirium screening and diagnosis with the Confusion Assessment Method (CAM) being one of the most used [82]. More recently a new tool, the 4AT has been developed to fill existing gap in the use of screening and diagnostic tools (Fig. 2) (https://www.the4at.com). In particular, the 4AT has important key features: it takes less than 2 min to be administered; it is suitable for use in normal clinical practice; it does not require specific training. It has been validated in geriatric and rehabilitation settings [50]. The 4AT can easily be used by all health care providers including nurses, physiotherapists and occupational therapists.

**Table 2:** DSM-5 and ICD-10 diagnosis of delirium

	DSM-5	ICD-10
Attention	Disturbance in ability to direct, focus, sustain, or shift attention.	Reduced ability to focus, sustain, or shift attention.
Awareness	Disturbance in awareness environmental orientation.	Clouding of consciousness, that is, reduced clarity of awareness of the environment.
Timing / Fluctuation	Develops quickly (hours to days) and represents a change from baseline and fluctuates over a day.	Rapid onset and fluctuations of the symptoms over the course of the day.
Memory Deficit	An additional disturbance in cognition (e.g. memory deficit, disorientation, language, visuospatial ability, or perception).	Disturbance of cognition, manifest by both: (1) impairment of immediate recall and recent memory, with relatively intact remote memory; (2) disorientation in time, place, or person.
Psychomotor Deficit	None	At least one of the following psychomotor disturbances: (1) rapid unpredictable shifts from hypoactivity to hyperactivity; (2) increased reaction time; (3) increased or decreased flow of speech; (4) enhanced startle reaction.
Sleep Disturbance	None	Disturbance of sleep or the sleep/wake cycle, manifest by at least one of the following: (1) insomnia, which in severe cases may involve total sleep loss, with or without daytime drowsiness, or reversal of the sleep/wake cycle; (2) nocturnal worsening of symptoms; (3) disturbing dreams and nightmares that may continue as hallucinations or illusions after awakening.
Corroborating Data	There is evidence from the history, physical examination or laboratory findings that the disturbance is a direct physiological consequence of another medical condition, substance intoxication or withdrawal, or exposure to a toxin, or is due to multiple etiologies.	Objective evidence from history, physical and neurological examination, or laboratory tests of an underlying cerebral or systemic disease (other than psychoactive substance-related) that can be presumed to be responsible for the clinical manifestations.
Other Cognitive Disorders	Not better explained by a pre-existing, established or evolving neurocognitive disorder and do not occur in the context of a severely reduced level of arousal, such as coma.	None

Since clinical observations of nurses play an important role, other instruments for bedside screening, monitoring and follow-up of patient behavior and cognitive functioning - such as the Delirium Observation Screening (DOS) Scale [52, 83] should be considered as well. Another scale might be easily used by the nurses as a delirium screening tool. The RADAR is a nursing screening scale that has been recently developed and it is based on the observation of the patients by the nurses during the drugs administration [84]. The ease of use and relevance for practice of such observational instruments; and the absence of response burden on patients make these kinds of instruments more feasible for implementation in daily care. If possible they should link to action and by this process become embedded with care processes in a manner that has the potential to empower clinical staff such as nurses who may not ordinarily be able to contribute to care [85].

Recent evidence has also underlined the importance of monitoring motor fluctuations in detecting for delirium. Motor fluctuations could be monitored using different tools such as the TCT and the HABAM [57–59]. A study comparing 4 groups of 15 patients (with delirium alone, with dementia alone, with delirium superimposed on dementia and with neither delirium nor dementia) found that when delirium develops, a worsening of motor performance - evaluated with the TCT- also occurs [65]. Motor performance returns to the previous status once delirium is resolved. Another investigation reported worse functional status as measured with the HABAM in patients with delirium and in particular in patients with delirium superimposed on dementia. This is a key message to spread in an interdisciplinary approach involving nurses, physiotherapists, and occupational therapists. In fact, these health care providers are in a good position to use this simple evaluation to screen for the possible presence of delirium and to proceed with a more formal evaluation with tools such as the 4AT and prompt a more formal geriatrics evaluation.

**Fig. 3:**
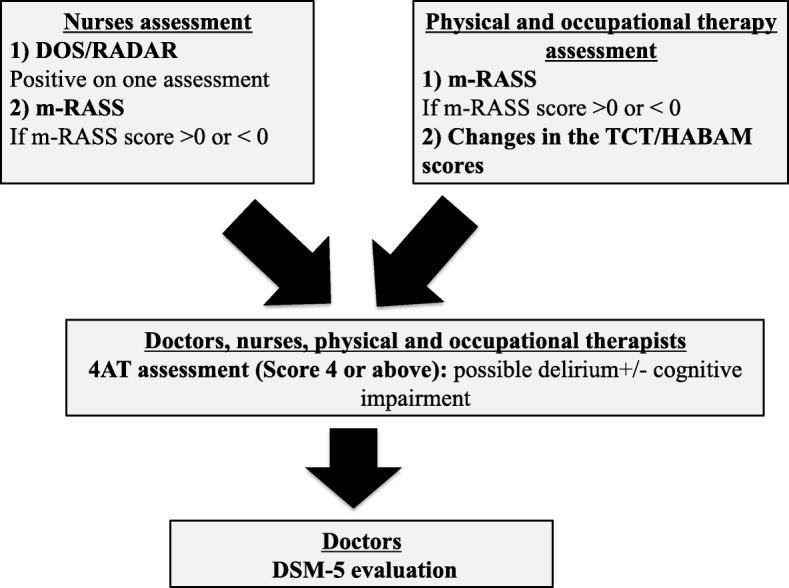
A multiprofessional approach to reduce underdetection of delirium. The nurses and physical/occupational therapists could screen patients for delirium on admission and on daily basis using different tools (i.e. Delirium Observation Scale, DOS; RADAR; modified Richmond Agitation and Sedation Scale, m-RASS; Trunk control Test, TCT; Hierarchical Assessment of Balance and Mobility, HABAM). If these evaluations are positive a second step approach including for instance the 4AT assessment should be performed followed by a DSM-5 evaluation for the confirmation of the presence of delirium

Here we can propose a multiprofessional approach to avoid the underdetection of delirium in the daily clinical practice and increase the team ability to flag high-risk patients (Fig. 3).

#### Key points for large-scale implementation of the interdisciplinary collaboration

It is know accepted that delirium programs are effective when a delirium champion is identified to promote the knowledge, education and implementation of delirium management [86]. The EDA is the leading scientific society on delirium in Europe. The EDA is a professional association providing a platform for practitioners, researchers, policy makers and other interested to network for the benefit of patients. The main goal of the EDA is to work on promoting delirium care with all those dedicated to delirium care and research, joining efforts to increase the disorder’s visibility not only in the general public, but also in the medical professional setting. Therefore, we believe the EDA should be leading the effort to promote the interdisciplinary collaboration among the scientific societies involved to identify delirium champions within each society. The role of each society is not only to endorse delirium knowledge and education among health care providers but also to increase awareness in the community and the healthcare stakeholders. Finally, it will be informative to collect information on the economic costs of such implementations since it has been shown that delirium programs can significantly reduce health care costs in patients with delirium [87].

## Conclusions

Delirium care should be based on an interdisciplinary, multi-dimensional diagnostic and therapeutic approach involving different health care professionals. However, there are still important gaps in the knowledge and management of this syndrome. In this paper we have strongly promoted and supported interdisciplinary collaboration underlying the necessity of increasing communication among scientific societies. We have also provided suggestions on how to fill the current gaps via improvements in undergraduate and postgraduate delirium education. The paper provides also a stimulus for researchers on the identification of possible ways on how to translate knowledge into practice, increase the education of health care providers on the topic of delirium and promote a more appropriate care for patients with delirium.

## Data Availability

Not applicable

## Abbreviations

ADL: Activities of daily living
CAM: Confusion Assessment Method
CGA: Comprehensive geriatric assessment
COPM: Canadian Occupational Performance Measure
COTEC: Council of Occupational Therapists for European Countries
DEMMI: De Morton Mobility Index
DOS: Delirium observation screening scale
DSM-5: Diagnostic and Statistical Manual of Mental Disorders-5
EANS: European Academy of Nursing Science
EDA: European Delirium Association
EuGMS: European Geriatric Medicine Society
HABAM: The Hierarchical Assessment of Balance and Mobility scale
HELP: Hospital Elder Life Program
ICU: Intensive Care Units
IPTOP/WCPT: International Association of Physical Therapists working with Older People of the World Confederation for Physical Therapy
OT: Occupational therapy
PT: Physical therapist
RASS: Richmond Agitation and Sedation Scale
SPPB: Shorth Physical Performance Battery
TCT: Trunk Control Test
WHO: World Health Organization
